# The association between intimate partner violence against women and newborn telomere length

**DOI:** 10.1038/s41398-019-0575-6

**Published:** 2019-09-30

**Authors:** Ko Ling Chan, Camilla K. M. Lo, Frederick K. Ho, Wing Cheong Leung, Benjamin K. Yee, Patrick Ip

**Affiliations:** 1 Department of Applied Social Sciences, The Hong Kong Polytechnic University, Hong Kong, China; 2 Institute of Health and Wellbeing, University of Glasgow, Glasgow, United Kingdom; 3 Department of Obstetrics & Gynaecology, Kwong Wah Hospital, Hong Kong, China; 4 Department of Rehabilitation Sciences, The Hong Kong Polytechnic University, Hong Kong, China; 5 Department of Paediatrics and Adolescent Medicine, The University of Hong Kong, Hong Kong, China

**Keywords:** Biomarkers, Psychology

## Abstract

Intimate partner violence (IPV) against women negatively impacts infant health. However, its impact on infant’s biology, in particular on telomere length (TL) is unknown. The aim of this study was to examine the association between IPV against women before childbirth and cord blood TL in their newborn. A total of 774 pregnant women in the 20th–24th week of gestation were recruited at a public hospital in Hong Kong. The mothers’ exposure to IPV before childbirth, demographic characteristics, obstetric outcomes, health and mental health were measured at the time of recruitment and 4 weeks after childbirth. Umbilical cord blood was collected by midwives at the time of delivery. The newborn TL was quantified using quantitative PCR method and expressed in T/S ratio (the ratio of telomere repeat copy numbers to single-copy gene numbers). After adjusting for a number of confounding variables, the mothers’ exposure to any IPV before childbirth (*β* = −0.08, 95% CI = −0.14, −0.01) was associated with shorter TL. Specifically, psychological abuse against women before childbirth (*β* = −0.08, 95% CI = −0.15, −0.02) and sexual abuse against women before childbirth (*β* = −0.22, 95% CI = −0.43 to −0.01) were significantly associated with reduced newborn TL. This study is the first to provide evidence of an association between IPV against women before childbirth and TL shortening in their newborns. Through TL- dependent transcription and epigenetic mechanisms, our finding suggests maternal exposure to IPV may exert a life-long impact on the offspring’s health.

## Introduction

Intimate partner violence (IPV) against women has substantial impacts on the women’s physical, mental, and reproductive health^1^. The negative impacts of IPV against women on infants health include preterm delivery, low birth weight, and a range of social and emotional problems^2^. Recently, the biological mechanisms related to transgenerational health risk as a result of IPV against women have attracted increasing attention. One line of investigation into the possible impacts of IPV at the cellular level has focused on telomere function, in particular through the examination of telomere length (TL). TL is a useful marker for telomere integrity, which is known to be sensitive to aging and may mediate some of the long-term adverse health outcomes in abused women^3^. However, the precise impacts of IPV against women on children’s TL has yet been unequivocally ascertained.

Telomeres are nucleoprotein complexes comprising repetitive DNA sequences -TTAGGG- and are located at the ends of the eukaryotic chromosomes, which serve to protect genomic integrity from damages caused by replication flaws^4^. Shorter TL has been linked to a higher risk of developing chronic diseases, such as type 2 diabetes, cancers, and cardiovascular diseases in adults^5,6^. Growing studies have pointed to the particular importance of TL dynamics in early stage of life than in adult life. A study has shown that the ranking of TL of most of the study participants did not change across their adult life^7^. Another study has shown a wide variation of TL across newborns but not within newborns^8^. These findings suggest that TL attrition is faster during early stage of life than in adulthood and that TL is largely determined before adulthood. Whilst TL shortening occurs natural as part of aging, heritability, and early life environment are the major determinants of TL^9^. One important advancement is the appreciation that TL erosion may result from childhood exposure to adversities, including childhood abuse and neglect^10^. However, these studies were predominantly conducted in adults, whose recalls of exposure to violence years after the events happened could be subject to bias. In addition, the TL obtained in adult subjects is likely also influenced by other confounding factors, such as lifestyle and engagement in health-compromising behaviors^11,12^.

The development and the health of children are interwoven with the well-being of their family. Children’s exposure to violence could be particularly detrimental to their long-term health given that the early development of multiple biological systems is highly sensitive to early life stressors and cumulative effects of these stressors^13^. A few studies have reported the association between children’s TL shortening and family problems, including family violence and marital conflict^14,15^. However, these studies have been challenged on methodological grounds, such as small sample size and reliance on a cross-sectional design. Thus far, Shalev et al. have provided the only longitudinal study involving 236 children. Those who experienced multiple types of violence, including domestic violence, child physical abuse, and bullying victimization, had significantly shorter TL in buccal DNA sampled from the age of 5–10 compared with children exposed to less or no violence^16^. However, still critically missing from the existing literature is an assessment of children’s TL at birth.

Recently, Enlow et al.^17^ were the first to report that maternal exposure to childhood sexual abuse before age 11 was related to TL shortening already at birth, particularly among boys. Their study suggested that variations of newborn TL may be associated with childhood abuse of the mother that may have exerted an effect in the intrauterine environment. In addition, observations linking newborn TL and other maternal factors, such as maternal demographic characteristics (e.g., maternal age and educational level^18,19^) and health and mental health factors (e.g., depression and stress) have been found^20,21^. Despite the indication of the link between newborn TL and maternal risk factors, maternal childhood abuse, and family problems, no study has specifically investigated the association between IPV against women and their offspring’s TL at birth. Hence, this study aimed to provide a more accurate estimation of the association between IPV against women and newborn TL by using a larger sample size, assessing child TL at birth using cord blood samples, and taking into consideration potential confounding factors in the analysis.

## Methods

### Participants

Participants were pregnant women recruited from the antenatal clinic of Kwong Wah Hospital, a public hospital managed by the Hospital Authority in Hong Kong. Of note, public hospitals in Hong Kong are highly subsidized and provide ~80% of in-patient services in the region. Kwong Wah Hospital has one of the city’s major obstetrics and gynaecology departments, providing services to ~5000 child births annually. During the study period (~3 months in 2017), we recruited 774 pregnant women who met the study’s inclusion criteria: (1) were age 18 years or above, (2) gave their informed consent, and (3) were in the 20th–24th week of gestation at the time of recruitment. At the time of recruitment, the mothers’ report of any history of exposure to IPV, demographic characteristics, health-related quality of life, engagement in risk behaviors, and mental health symptoms such as depression, stress, and anxiety were collected. Umbilical cord blood samples from the newborns were subsequently collected by midwives during delivery. The cord blood samples were used for assessing newborn TL. The mothers were contacted 4 weeks after delivery. The mothers reported again about their history of exposure to IPV at any time in their lives up to childbirth and their obstetric outcomes.

### Measures

#### Outcome measure

##### Newborn telomere lengths

Samples of the newborns’ DNA were extracted from the umbilical cord blood collected by midwives during each baby’s delivery. Genomic DNA samples were isolated and extracted from the collected samples using the QIAamp DNA Mini kit (Qiagen), according to the manufacturer’s instructions. Isolated DNA samples were eluted to the buffer solution (10 mM Tris-HCl and 1 mM ethylenediamine-tetraacetic acid, pH 8.0) for quality checking and quantification by spectrophotometry (NanoDrop 2000c, Thermo Scientific). Upon confirming that the DNA quality and quantity were in the acceptable range for the determination of telomere length, each sample was processed following the procedures described in previous literature^22^. Each sample was handled in triplicate for the telomere length assay by quantitative polymerase chain reaction (qPCR) using a 7900HT Thermocycler (Applied Biosystems). After the assay, the telomere length was presented by a relative ratio of the telomere repeat copy number (T) to single-copy gene 36B4 copy number (S) using the formula of T/S = 2^(−ΔCt)^, where ΔCt is the mean difference between the threshold cycle (Ct) value of the 36B4 gene and telomere repeats obtained from the qPCR performed.

#### Predictor

##### IPV against women by current partners before childbirth

The mothers reported on their exposure to violence by their current partners in the form of psychological, physical, and sexual abuse at any time in their lives up to childbirth, using the five-item Abuse Assessment Screen (AAS)^23^. The AAS has been commonly used in different healthcare settings, and the validated Chinese AAS demonstrated satisfactory measurement accuracy for identifying IPV against women when it was used to evaluate Chinese women in a previous study^24^. The mothers who reported any history of exposure to IPV at any time in their lives up to the time of childbirth were categorized into “abused group” and those who did not have any history of exposure to IPV were categorized into “non-abused group.”

#### Covariates

##### Maternal anxiety and stress symptoms

These were assessed using the anxiety and stress subscales of the Chinese version of the Depression Anxiety Stress Scale (DASS)^25^. The DASS has been widely used in research and clinical settings. The anxiety and stress subscales each consist of seven items that assess the severity (identified as normal, mild, moderate, and severe) of respective core symptoms. The DASS has been validated in a Chinese population^26^. The symptom scores were adjusted in the regression models as continuous variable.

##### Maternal depressive symptoms

Maternal depressive symptoms were assessed using the 10-item Edinburgh Postnatal Depression Scale (EPDS)^27^. The EPDS assesses the presence of maternal depressive symptoms during the perinatal period. The mothers were asked to report the presence of depressive symptoms. The Chinese version of EPDS has been validated in a previous study^28^. The symptom scores were adjusted in the regression models as a continuous variable.

##### Maternal health-related quality of life

The Chinese version of the Short-Form Health Survey, version 2 (SF-12)^29^ was used to assess the mothers’ maternal health-related quality of life (HRQoL). The SF-12 consists of 12 items that measure eight health domains, including physical functioning, role limitations due to physical health, bodily pain, general health, vitality, social functioning, role limitations due to emotional health, and mental health. The SF-12 generates two composite scores, the physical component summary (PCS) and the mental component summary (MCS), which were adjusted in the regression models as continuous variables.

##### Maternal risk behaviors

Maternal risk behaviors, including gambling, cigarette smoking, alcohol drinking, and drug use were assessed using four items. The mothers were asked to report how often they engaged in each of those risk behaviors, by rating them as 1 = never; 2 = rarely; 3 = sometimes; and 4 = always. Because these risk behaviors were not common in this sample, the variables were grouped as binary variables (never vs. ever engaged in the behavior) in the analysis.

##### Demographic characteristics and obstetric factors

Demographic information about the mothers, such as maternal age, educational level, marital status, family income, self-reported obstetric problems (hyperemesis gravidarum, hypertension, diabetes mellitus, multiple pregnancy, and vaginal bleeding), gestation age, and birth weight were collected and considered in our analyses. Maternal age, family income, gestation age, and birth weight were adjusted as continuous variables, while education level, marital status, and obstetric problems were adjusted as categorical variables.

##### Data analysis

Descriptive statistics of the participants’ characteristics and exposure to IPV before childbirth were first computed. To examine the association between IPV against the women before childbirth and TL in their newborns, four regression models were generated with increasing number of covariates to examine the influence of adjustment covariates on the association. This approach is to illustrate the robustness of the association regardless of adjustment schemes, rather than for variable selection as in traditional stepwise approach. The statistical significance of the adjustment variables is not of primary interest in this study and serve as a reference only. The first model was of crude associations between different types of IPV against the women before childbirth and their newborns’ TL. In the second model, the associations were examined with adjustments for demographic variables, obstetric problems, and newborn characteristics. The third model additionally included the mothers’ health-related quality of life as the adjusted variables. The fourth model additionally included the mothers’ mental health variables as the adjusted variables. These adjustment variables were selected due to previous reports on their simultaneous association with the exposure (IPV) and outcome (TL). Due to a highly positive skewness of the distribution (2.34), a logarithmic transformation of the TL was performed before the analysis. Data analyses were performed using R Statistical Software. All tests were two-tailed, and *P*-values below 0.05 were regarded as statistically significant. Multicollinearity was examined using variance inflation factor (VIF) where variables with VIF >10 were removed as a sensitivity analysis.

##### Ethical approval

The research protocol was approved by the Institutional Review Board of the Hospital Authority Kowloon West Cluster Research Ethics Committee (Reference number: KW/FR-16-042(97-01)(1)).

## Results

As Table 1 shows, the mean age of the mothers in this study was 31.05 years (SD = 4.5). The majority of them (92.1%) were married, and 7.9% were single or divorced. More than half (63.3%) of them had obtained a post-secondary education or above. The mean monthly family income was equivalent to USD 4827 (SD = 3188). The mothers in the abused group had higher levels of depressive, anxiety, and stress symptoms, compared to the mothers in the non-abused group (*p*-values < 0.0001). The mothers in the non-abused group were more likely than the mothers in the abused group to have hypertension (Fisher’s exact test = 4.88, *p* = 0.02) and ever drunk alcohol (Fisher’s exact test = 7.65, *p* = 0.006). In regard to the infants, the mean age of gestation was 38.73 weeks (SD = 1.43). The mean birth weight was 3074.28 grams (SD = 553.08). The mean telomere length (T/S) was 15.12 (SD = 6.34) and it was significantly shorter in the abused group (Mean = 14.26, SD = 5.54) than the non-abused group (Mean = 15.39, SD = 6.54), *t*(772) = 4.44, *p* < 0.04. The mean T/S ratio measured in this study is similar to that reported by a previous study in children (with a median T/S ratio of 10.87 ± 6.4)^30^. Table 2 shows the percentages of the women who were exposed to different types of IPV by their current partners. Nearly one-quarter (23.5%) of the mothers in this study reported exposure to IPV by their current partners before childbirth. Psychological abuse was the most common type of abuse (23.3%), followed by physical abuse (3.5%) and sexual abuse (1.8%). Among the women in this study, 18.9% were exposed to one type of IPV, 4.3% were exposed to two types of IPV, and 0.4% were exposed to three types of IPV.

**Table 1:** Characteristics of study participants

	All (*N* = 774)	Non-abused (*N* = 592)	Any abuse (*N* = 182)	Chi-squared-/*t*-statistic (df)	*P*-value
Mean (SD) maternal age at recruitment	31.05 (4.50)	31.24 (4.40)	30.44 (4.78)	4.40 (772)	0.04
Marital status				2.76 (1)	0.10
Single/divorced	60 (7.9)	40 (6.9)	20 (11.1)		
Married	698 (92.1)	538 (93.1)	160 (88.9)		
Maternal educational level				8.78 (4)	0.07
Junior secondary school or below	61 (7.9)	44 (7.4)	17 (9.3)		
Senior secondary school	223 (28.8)	176 (29.7)	47 (25.8)		
Post-secondary	91 (11.8)	77 (13.0)	14 (7.7)		
Bachelor’s degree	151 (19.5)	105 (17.7)	46 (25.3)		
Postgraduate degree	248 (32.0)	190 (32.1)	58 (31.9)		
Mean (SD) family monthly income (USD)	4827.35 (3188.22)	4792.08 (3136.56)	4939.51 (3353.99)	0.29 (738)	0.59
Mean (SD) gestation age (weeks)	38.73 (1.43)	38.71 (1.39)	38.79 (1.54)	0.30 (457)	0.58
Mean (SD) birth weight (g)	3074.28 (553.08)	3074.07 (535.60)	3074.88 (601.69)	0.00 (454)	0.99
Mean (SD) telomere length (T/S)	15.12 (6.34)	15.39 (6.54)	14.26 (5.54)	4.44 (772)	0.04
Mean (SD) SF-12 PCS	46.19 (6.82)	46.30 (6.87)	45.81 (6.64)	0.72 (772)	0.40
Mean (SD) SF-12 MCS	48.42 (8.67)	49.39 (8.27)	45.24 (9.19)	33.26 (772)	<0.0001
Obstetric problems					
Hyperemesis gravidarum	17 (2.2)	15 (2.5)	2 (1.1)	0.75 (1)	0.39
Hypertension	19 (2.5)	10 (1.7)	9 (4.9)	4.88 (1)	0.02
Diabetes mellitus	56 (7.2)	40 (6.8)	16 (8.8)	0.58 (1)	0.45
Multiple pregnancy	5 (0.6)	4 (0.7)	1 (0.5)	0.00 (1)	1.00
Vaginal bleeding	27 (3.5)	21 (3.5)	6 (3.3)	0.00 (1)	1.00
Ever gambled	255 (32.9)	190 (32.1)	65 (35.7)	0.67 (1)	0.41
Ever smoked	90 (11.6)	66 (11.1)	24 (13.2)	0.38 (1)	0.54
Ever drunk alcohol	232 (30.0)	162 (27.4)	70 (38.5)	7.65 (1)	0.006
Ever use drugs	2 (0.3)	2 (0.3)	0 (0.0)	–	–
Mean (SD) depressive symptom score	7.15 (4.63)	6.68 (4.44)	8.67 (4.92)	26.44 (772)	<0.0001
Mean (SD) anxiety symptom score	5.63 (5.15)	5.21 (4.89)	7.00 (5.71)	17.25 (772)	<0.0001
Mean (SD) stress symptom score	7.43 (6.77)	6.64 (6.44)	10.01 (7.18)	36.16 (772)	<0.0001

Numbers presented are *n* (%) unless otherwise specified. Tests for difference between non-abused and any abuse groups were chi-square test/Fisher exact test for independence (categorical variables) and independent *t*-test (continuous variables)

**Table 2:** Participants’ exposure to IPV before childbirth

Type of IPV before childbirth	All (*N* = 774) *n* (%)
Psychological abuse	180 (23.3)
Physical abuse	27 (3.5)
Sexual abuse	14 (1.8)
Any abuse	182 (23.5)
Number of types of abuse	
0	592 (76.5)
1	146 (18.9)
2	33 (4.3)
3	3 (0.4)

Table 3 shows the results of regression analyses on the associations between IPV against women by their current partners before childbirth and their newborns’ TL. The first regression analysis model showed the crude associations between different types of IPV against women by their current partners before childbirth and newborn TL. Any abuse (*β* = −0.08, 95% CI = −0.14 to −0.02, *p* = 0.01), psychological abuse (*β* = −0.08, 95% CI = −0.14 to −0.02, *p* = 0.01) and sexual abuse (*β* = −0.19, 95% CI = −0.39 to 0.00, *p* = 0.048) of the mothers were significantly associated with the logarithm of their newborns’ TL. Physical abuse was not significantly associated with newborn TL. In the final model, after adjusting for a number of confounding variables, such as gestation age, birth weight, maternal age, educational level, family income, marital status, obstetrics problems, newborn characteristics, and maternal health and mental health factors, any IPV by current partners before childbirth (*β* = −0.08, 95% CI = −0.14, −0.01, *p* *=* 0.02) was associated with the logarithm of the TL in the newborns. Specifically, psychological abuse (*β* = −0.08, 95% CI = −0.15, −0.02, *p* = 0.01) and sexual abuse (*β* = −0.22, 95% CI = −0.43 to −0.01, *p* = 0.04) before childbirth were significantly associated with the newborns’ TL, which was shorter by 21.4% and 61.5% of standard deviation of the log-transformed TL, respectively. Mental health-related variables showed strong inter-correlation (VIFs ranged 2.25–2.75) but there was no evidence for severe multicollinearity (all VIFs ≤ 2.75).

**Table 3:** Associations between IPV against women by their current partners before childbirth and their newborns’ TL

	Model 1: Crude associations		Model 2: IPV, Controlled for maternal demographics, obstetric problems, and newborn characteristics		Model 3: IPV, Controlled for demographics, health variables		Model 4: IPV, Controlled for demographics, health variables, and mental health variables	
	*β* (95% CI)	*P*	*β* (95% CI)	*P*	*β* (95% CI)	*P*	*β* (95% CI)	*P*
IPV before childbirth								
Psychological abuse	−0.08 (−0.14, −0.02)	0.01	−0.08 (−0.14, −0.02)	0.01	−0.09 (−0.15, −0.02)	0.009	−0.08 (−0.15, −0.02)	0.01
Physical abuse	0.00 (−0.14, 0.14)	0.95	−0.06 (−0.20, 0.09)	0.45	−0.06 (−0.21, 0.09)	0.40	−0.06 (−0.21, 0.09)	0.42
Sexual abuse	−0.19 (−0.39, 0.00)	0.048	−0.22 (−0.42, −0.02)	0.03	−0.24 (−0.44, −0.03)	0.02	−0.22 (−0.43, −0.01)	0.04
Any abuse	−0.08 (−0.14, −0.02)	0.01	−0.08 (−0.14, −0.01)	0.02	−0.08 (−0.15, −0.02)	0.01	−0.08 (−0.14, −0.01)	0.02
Covariates								
Gestation age			0.00 (0.00, 0.00)	0.27				
Birth weight			−0.01 (−0.03, 0.02)	0.69				
Hyperemesis gravidarum			−0.19 (−0.38, −0.01)	0.04				
Hypertension			−0.01 (−0.19, 0.17)	0.9				
Diabetes mellitus			0.02 (−0.09, 0.12)	0.78				
Multiple pregnancy			0.66 (0.29, 1.04)	0.0006				
Vaginal bleeding			0.05 (−0.10, 0.19)	0.51				
Maternal age			0.01 (0.00, 0.01)	0.11				
Married			−0.06 (−0.16, 0.05)	0.28				
Family income			−0.02 (−0.05, 0.01)	0.16				
Education level								
Junior secondary			−0.03 (−0.17, 0.10)	0.62				
Senior secondary			−0.02 (−0.13, 0.09)	0.73				
Post-secondary			−0.08 (−0.20, 0.03)	0.16				
Bachelor’s degree			0.00 (−0.11, 0.11)	0.99				
Postgraduate degree			0 (Reference)					
SF-12 PCS					−0.02 (−0.05, 0.01)	0.21		
SF-12 MCS					0.00 (−0.03, 0.03)	0.95		
Ever gamble					−0.03 (−0.09, 0.03)	0.38		
Ever smoke					0.04 (−0.05, 0.13)	0.41		
Ever drink alcohol					0.00 (−0.07, 0.06)	0.91		
Ever use drugs					0.00 (−0.51, 0.52)	0.99		
Depressive symptoms							0.01 (−0.03, 0.05)	0.54
Anxiety symptoms							−0.01 (−0.05, 0.03)	0.61
Stress symptoms							−0.03 (−0.08, 0.01)	0.13

## Discussion

### Main findings

Despite a wealth of research showing that early life adversities such as violence can negatively affect health and morbidity^13^, little is known about the potential biological impacts of such adversities at the cellular level. This study provided the first evidence that IPV against women by current partners before childbirth was associated with TL shortening in their children that could already be detected at birth. This finding extends the concept of biological embedding^31^ by showing that the mother’s exposure to violence is important in understanding the newborn’s telomere biology.

In this study, approximately a quarter of women (23.5%) were exposed to IPV by their current partners before childbirth, with psychological abuse being the most common type of abuse (23.3%), followed by physical abuse (3.5%) and sexual abuse (1.8%). The pattern is comparable to previous findings on IPV in Chinese couples^32^. After controlling for a number of confounding factors, including maternal age, educational level, family income, obstetric problems, obstetric outcomes, health-related quality of life, engagement in risky behaviors (e.g., gambling, smoking, alcohol drinking, and substance use), depression, anxiety, and stress symptoms, IPV against women by current partners before childbirth was a significant predictor of the TL of the women’s newborns. Specifically, psychological abuse and sexual abuse perpetrated by a partner on a woman before childbirth were significantly associated with newborn TL shortening. However, this study did not find a significant association between physical abuse to the mothers and their newborns’ TL, suggesting psychological and sexual abuse may be more important than physical abuse in explaining newborn TL shortening. This explanation is consistent with previous studies that found robust and unique effects of psychological IPV on mental health, after controlling for the effect of physical IPV^33^, and a stronger effect of sexual IPV than physical IPV on depression^34^.

The association between IPV against women and TL in their newborns in this study raises the question of what possible biological mechanisms could explain such an association. The developing fetus is particularly sensitive to intrauterine influences^35^. One of the possible mechanisms to explain the findings of this study is the maternal-placental-fetal neuroendocrine processes^36^. Exposing mothers to a violent experience heightens their stress levels, and that added stress may in turn transmit from the mothers to the fetus through the release of maternal and placental hormone including cortisol and placental corticotropin-releasing hormone that may induce changes in placental physiology, which may in turn influence TL of the offspring. However, the contribution of maternal stress as a potential mediator of the reported associations here warrant further investigation.

### Strengths

One of the methodological strengths of this study is the use of a relatively large sample (*N* = 774) of mother-infant dyads, particularly for a study of cord blood TL. In addition, the use of cord blood TL permitted us to assess the impact of pre-birth factors on TL by minimizing the postnatal environmental confounding factors. As maternal factors may also influence newborn TL, this study statistically adjusted for a number of confounding variables related to maternal demographic characteristics, newborn characteristics, obstetric problems, maternal health and mental health.

### Limitations

The study sample consisted solely of pregnant women recruited at a single antenatal clinic of a public hospital in Hong Kong. This sampling method limited the external generalizability of the findings to the rest of the population. The use of self-report measurements of IPV against women was subject to several limitations, such as participants’ social desirability in responding, and their recall bias, which might result in underestimation of the violence. Also, this study did not examine the father’s characteristics and lacked data on infant sex, which may have an influence on the newborn TL. Another limitation is that this study only assessed TL in venous cord blood and was unable to adjust for cell types in the analyses. Although TL in different tissues such as white blood cell, foreskin and umbilical artery within the newborn are highly correlated^8^, further study to include TL in different cell types is needed to validate whether cell type is important in understanding the impact of IPV against women on newborn TL.

### Implications

Human TL is largely determined during the first two decades of life and to a large degree at birth^7^. This study advanced our knowledge that IPV against women is a relevant prenatal predictor of newborn TL shortening. Although existing evidence suggests that TL is a biological marker for various chronic diseases in adults^5,6^, the link between TL shortening and pediatric health has been less consisent^37,38^. Also, the causal roles of TL in diseases have yet to be established. Longer-term longitudinal investigations to track children’s TL from birth through early childhood, using multiple measurement points and including a comprehensive family profile of exposure to violence, would shed more light on the complexity of exposure to violence and any subsequent changes in the TL of children during their developmental years.

In addition, a previous study showed that IPV against pregnant women is associated with subsequent risk of child maltreatment^39^ and such intergenerational abuse will further jeopardize children’s health and well-being. Moreover, consistent evidence has shown a dose-response relationship between exposure to IPV and various outcomes in victims and their children^40,41^. Prolonged and severe violence may moderate intergenerational transmission of abuse and the effect of IPV against women on newborn TL. Hence, health professionals should routinely screen for IPV against women to safeguard the overall well-being of children and women. In particular, pregnancy is a period that offers an opportunity for health professionals to screen for pregnant women’s exposure to IPV. Although IPV in form of psychological abuse does not leave visible wounds on the victims, its impact on abused women’s newborns is significant. More attention should be given to this form of IPV. Providing psychological services and parenting support for mothers exposed to IPV may be helpful in buffering the long-term effects of IPV on child health, as positive parenting has been found to potentially decrease the rate of postnatal TL shortening in children^42^.

## Conclusions

This study demonstrates an association between IPV against women by their current partners before childbirth and their newborns’ biology, in the form of TL shortening, even after controlling for a number of confounding factors. This finding extends the concept of biological embedding by showing that the mother’s exposure to violence is a relevant predictor of newborn’s telomere biology.
